# A Novel Strategy Conjugating PD-L1 Polypeptide With Doxorubicin Alleviates Chemotherapeutic Resistance and Enhances Immune Response in Colon Cancer

**DOI:** 10.3389/fonc.2021.737323

**Published:** 2021-11-10

**Authors:** Maolin Wang, Xing-sheng Shu, Meiqi Li, Yilin Zhang, Youli Yao, Xiaoyan Huang, Jianna Li, Pengfei Wei, Zhendan He, Jun Lu, Ying Ying

**Affiliations:** ^1^ Department of Physiology, School of Basic Medical Sciences, Shenzhen University Health Science Center, Shenzhen, China; ^2^ College of Pharmacy, Shenzhen Technology University, Shenzhen, China; ^3^ Department of Pathogen Biology, School of Basic Medical Sciences, Shenzhen University Health Science Center, Shenzhen, China; ^4^ Shenzhen University General Hospital, Department of Endocrinology, Shenzhen, China; ^5^ School of Pharmacy, Chengdu University of Traditional Chinese Medicine, Chengdu, China; ^6^ Institute of Integrated Bioinfomedicine & Translational Science, Hong Kong Baptist University Shenzhen Research Institute and Continuing Education, Shenzhen, China

**Keywords:** target delivery system, colon cancer, PD-L1 targeting polypeptide, pH sensitive linker, chemotherapeutics drug release

## Abstract

**Background:**

Modifying the structure of anti-tumor chemotherapy drug is of significance to enhance the specificity and efficacy of drug-delivery. A novel proteolysis resistant PD-L1-targeted peptide (PPA1) has been reported to bind to PD-L1 and disrupt the PD-1/PD-L1 interaction, thus appearing as an outstanding tumor-targeting modification of synergistic drug conjugate for effective anti-tumor treatment. However, the combination regimen of coupling PD-L1 polypeptide with chemotherapeutic drug in tumoricidal treatment has not been reported thus far.

**Methods:**

We developed a novel synergistic strategy by conjugating PPA1 to doxorubicin (DOX) with a pH sensitive linker that can trigger the release of DOX near acidic tumor tissues. The binding affinity of PPA1-DOX with PD-L1 and the acid-sensitive cleavage of PPA1-DOX were investigated. A mouse xenograft model of colon cancer was used to evaluate the biodistribution, cytotoxicity and anti-tumor activity of PPA1-DOX.

**Results:**

PPA1-DOX construct showed high binding affinity with PD-L1 *in vitro* and specifically enriched within tumor when administered *in vivo*. PPA1-DOX exhibited a significantly lower toxicity and a remarkably higher antitumor activity *in vivo*, as compared with free PPA1, random polypeptide-DOX conjugate, DOX, or 5-FU, respectively. Moreover, increased infiltration of both CD4^+^ and CD8^+^ T cells was found in tumors from PPA1-DOX treated mice.

**Conclusions:**

We describe here for the first time that the dual-functional conjugate PPA1-DOX, which consist of the PD-L1-targeted polypeptide that renders both the tumor-specific drug delivery and inhibitory PD-1/PD-L1 immune checkpoint inhibition, and a cytotoxic agent that is released and kills tumor cells once reaching tumor tissues, thus representing a promising therapeutic option for colon cancer with improved efficacy and reduced toxicity.

## 1 Introduction

Chemotherapy is one of the major categories of the medical discipline specifically devoted to pharmacotherapy for variety of cancers, including colon cancer (1, 2). Since the anti-tumor drugs of chemotherapy do not distinguish tumor cells from normal tissue cells, chemotherapeutic techniques have a range of undesirable side effects (3). Modifying the structure of anti-tumor chemotherapy drug is of significance, which allows the drug to recognize tumor cells and reduce the lethality to normal tissue cells. There have been several techniques that attach the targeted recognition part to chemotherapy drugs. Antibody-drug conjugate (ADC) is a class of biopharmaceutical drugs combining antibodies with chemotherapy drugs, however, the risk of immunogenicity and the raising incidents of resistance still limit its clinical treatment (4–6). Aptamer drug conjugate (ApDC) is another class of molecule that binds to a specific target protein (7, 8). ApDCs are comprised of targeted component and drug component. Generally, the nucleic acid and polypeptide are utilized to bind to a specific target, such as nucleolin, EGFR and Vimentin for tumor cells (9–11). However, this approach has suffered from rapid elimination by systemic clearance. Therefore, it is urgently desirable to develop a tumor targeting modification method to enhance drug-delivery efficacy and reduce side effects.

Target polypeptides are artificial proteins selected or engineered to bind specific target molecules, which consist of a number of peptides forming loops of variable sequence and displaying unique protein scaffold (12). To prevent the degradation of proteolytic enzymes, D-polypeptide was chosen as the target polypeptide. Programmed death-ligand 1 (PD-L1) has been proven to play a major role in suppressing the activity of T cells of immune system and up-regulated in various types of cancers (13–15). The blockade of PD-L1 by target polypeptides could disrupt the inhibitory PD-1/PD-L1 immune checkpoint and provide a promising cancer treatment (16, 17). A novel polypeptide PPA1 has been reported that it can bind PD-L1 *in vitro* and inhibit the tumor growth in CT26 bearing mice by disrupting the PD-1/PD-L1 interaction. The D-peptide construct of PPA1 may prevent the degradation of proteolytic enzymes in serum (18). Therefore, PPA1 appears as an outstanding tumor-targeting modification of synergistic drug conjugate for effective anti-tumor treatment. However, the combination regimen of coupling PD-L1 polypeptide with chemotherapeutic drug in tumoricidal treatment has not been reported thus far.

Studies have shown that intracellular pH of solid tumors is maintained in a range of 7.0 to 7.2, whereas the extracellular pH demonstrates acidic microenvironment (19). The acidic microenvironment may be a significant factor that could trigger the release of the anti-tumor chemotherapeutic drug in tumor tissues (20), but to keep the chemotherapeutic construct steady in non-tumor tissues. Therefore, the polypeptide and drug can be conjugated by an acid-sensitive linker, forming a polypeptide-drug conjugate that is able to stay steady in normal tissue and be specifically delivered to tumor tissue by target polypeptide, then release chemotherapeutic drug due to the cleavage of acidic pH sensitive linker.

In this study, the proteolysis resistant PD-L1-targeted peptide, PPA1, was conjugated to doxorubicin (DOX) with a pH sensitive linker. The reason that we did not select 5-Fu as the conjugated drug was the lack of suitable synthetic site on 5-Fu. Although the development of resistance to DOX in colon cancer has been shown (21), DOX was selected as the candidate chemotherapeutic drug herein to verify the feasibility of reducing tumor drug-resistance by improving tumor-specific targeted drug delivery. We found that PPA1-DOX construct showed high binding affinity with PD-L1 *in vitro* and was specifically enriched within tumor when administered *in vivo*. Moreover, a significantly lower toxicity and higher antitumor activity was achieved by PPA1-DOX *in vivo*, as compared with the respective free PPA1, random polypeptide-DOX conjugate, DOX, or 5-FU. Thus, we believe that the dual-functional conjugates, which consist of the PD-L1-targeted polypeptide that renders both the tumor-specific drug delivery and inhibitory PD-1/PD-L1 immune checkpoint inhibition, and a cytotoxic agent that kills tumor cells once reaching tumor tissues, represents a promising therapeutic option for colon cancer.

## 2 Material and Methods

### 2.1 Synthesis Information

The PPA1 (nyskptdrqyhfk) and RNA (rhtndysqfypk) were purchased from Chinapeptides Co.,Ltd., China. The polypeptides were both in D-form.

Methanol, ethanol, trifluoroethanoic acid (TFA) and N,N-dimethylformamid (DMF) were purchased from Sigma-Aldrich. N,N’-dicyclohexylcarbodimide (DCC), 4-dimethylaminopyrid (DMAP), hydrazine hydrate (N_2_H_4_, about 80% in H_2_O), doxoriubicin, 4A molecular sieves, CuSO_4_.5H_2_O, sodium ascorbate, 1-(1-benzyltriazol-4-yl)-N,N-bis[(1-benzyltriazol-4-yl)methyl]- methanamine (TBTA) were purchased from TCI. DOX and 5-Fu were purchased from J&K Scientific Ltd. Those custom peptides of R(2-Azido) were synthesized by ChinaPeptides Co,. Ltd. All of the purchased chemicals were of at least reagent grade and were used without further purification. Reactions were monitored by analytical thin-layer chromatography (TLC) using silica gel 60 F254 pre-coated glass plates (0.25 mm thickness) and visualized using UV light.

The ^1^H and ^13^C NMR spectra were recorded on a Bruker Avance 400 MHz (^1^H: 400 MHz, ^13^C:101 MHz) spectrometer using tetramethylsilane (TMS) as internal standard at 25°C. Samples were prepared as solutions in deuterated solvent. Those following abbreviations were used to indicate the observed spin multiplicities on NMR spectra: s = singlet, d = doublet, t = triplet, q = quartet, dd = doublet of doublets, m = multiplet, and br = broad. High resolution mass spectra (HRMS) were recorded on Bruker Autoflex MALDI-TOF mass spectrometer. Purity of all final compounds was 95% or higher as determined by high performance liquid chromatography (HPLC) (SHIMADZU Labsolutions) analysis on the Aglilent C18 column (4.6 × 250 mm, 5 μm) using gradient elution (Mobile Phase: A Phase = ACN, B Phase = 0.3% H_3_PO_4_ in H_2_O) at a flow rate of 1.0 mL/min.

#### 2.1.1 Synthesis of Methyl hex-5-ynoate (2)

To a solution of 5-hexynoic acid (2.00 g, 17.85 mmol) in MeOH (30 mL), DCC (3.67 g, 17.85 mmol) and DMAP (2.40 g,19.64 mmol) were added successively and the mixture was stirred at room temperature for 4 h, then filtrated and concentrated under reduced pressure. Weak acidic water was added and extracted with EtOAc (4 * 30 mL). The organic layers were dried over Na_2_SO_4_ and the solvent was removed under reduced pressure. The residue was purified by column chromatography to give **2** (1.98 g, yield 88%) as a colorless oil.

#### 2.1.2 Synthesis of Hex-5-ynehydrazide (3)

To the solution of **2** (1.26 g, 10 mmol) in EtOH (30 mL) was added 80% hydrazine hydrate solution in H_2_O (1 mL) at room temperature. Then the mixture was stirred at 80°C for 6 h and the solution was evaporated *in vacuo*. The residue was dissolved in EtOAc and washed with aqueous citric acid (*3) and brine (*2). The organic solution was dried over Na_2_SO_4_, filtered and evaporated *in vacuo*. The residue was purified by column chromatography to obtain **3** (1.02 g, yield 81%) as a yellow solid.

#### 2.1.3 Synthesis of Hydrazine-DOX (4)

Doxorubicin hydrochloride (1.56 g, 2.7 mmol) and **3** (0.38 g, 3.0 mmol) was dissolved in MeOH (30 mL) and treated with 4A molecular sieves following a drop of TFA (20 μL). The resulting mixture was stirred at room temperature for 24 h. Then the solvent was evaporated and the crude product was purified by column chromatography to provide **4** (1.12 g, yield 64%) as a reddish-brown solid.

#### 2.1.4 Synthesis of PPA1-DOX (5)

**4** (65.1 mg, 0.1 mmol), azide end-functionalized peptide (PPA1) (102.8 mg, 0.11 mmol) and Tris(benzyltriazolylmethyl)amine (TATB) (10.6 mg, 0.02 mmol) were introduced in a Schlenk tube and 4 mL of DMF: H_2_O (v: v = 3:1) were added. The solution was degassed by bubbling argon for 10 min. CuSO_4_.5H_2_O (5.0 mg, 0.02 mmol) and sodium ascorbate (4.0 mg, 0.02 mmol) were added to the mixture contained in the Schlenk tube and the mixture was degassed once more by bubbling argon for 10 min. The Schlenk tube was filled with argon and stirred at room temperature for 4 h. The solution was filtered and concentrated under vacuum. The resulting crude mixture was purified by HPLC to offer **5** (95.9 mg, yield 43%) as a reddish-brown solid.

#### 2.1.5 Synthesis of RNA-DOX (6), RhB-PPA1-DOX (7) and RhB-RNA-DOX (8)

The conjugates of **6**, **7** and **8** were prepared as the synthetic procedure of conjugate **5**.

### 2.2 Cell Culture

Mouse colorectal cancer cell line CT26 was purchased from Shanghai Cell Bank of Chinese Academy of Sciences. The cells were maintained in RPMI-1640 medium, supplemented with 10% fetal bovine serum, 1% penicillin-streptomycin, and incubated at 37°C with 5% CO_2_ and 95% humidity. The cells were cultured in T75 culture flask and the cell density up to 80% was used in experiments. The cell line was negative for mycoplasma.

### 2.3 Simulation of Docking Calculation and Molecular Dynamics

The three-dimensional models of PD-L1 were downloaded from Protein Data Bank (PDB) (PDB ID: 3BIK). The structure of the peptides was generated by Chimera 1.14. The molecular dynamic for the coarse structures were implemented for energy minimization and optimization in amber force field. Molecular docking was performed to generate the initial complex of PPA1-DOX or PPA1 and PD-L1 by using Cluspro 2.0 web server. The binding free energy was calculated with MM-PBSA algorithm.

### 2.4 PD1/PD-L1 Binding Assay

The interaction between Tag1-PD-L1 and Tag2-PD1 is detected by using anti-Tag1-Europium (HTRF donor) and anti-Tag2-XL665 (HTRF acceptor). When the donor and acceptor antibodies are brought into close proximity due to PD-L1 and PD1 binding, excitation of the donor antibody triggers fluorescent resonance energy transfer (FRET) towards the acceptor antibody, which in turn emits specifically at 665 nm. This specific signal is directly proportional to the extent of PD1/PD-L1 interaction. Thus, compound or antibody blocking PD1/PD-L1 interaction will cause a reduction in HTRF signal. The HTRF PD1/PD-L1 binding assay kit was from Cisbio. The IC_50_ is calculated by fitting the dose-response data to a sigmoidal curve, typically using the Hill equation. The calculation is performed by using ECCpy (https://github.com/teese/eccpy).

### 2.5 The Cleavage Assay for the PPA1-DOX

To verify the acid sensitivity of hydrazone bond linking the PPA1 to DOX, a series of PPA1-DOX and DOX solutions with different concentrations were configured in the sodium phosphate buffer (pH= 9.0), respectively. A 5 μL aliquot of each sample was injected onto an HPLC system with ultraviolet detector wavelength to determine 254 nm absorbance values, using hydrophilic interaction chromatography (HILIC) column separation (Waters, XBridge BEH HILIC XP Column, 2.1 mm, 50 mm, 2.5 µm). Through simulating the linearity fitting from disposed concentrations and detected absorbance values, standard curves of PPA1-DOX and DOX were obtained, respectively.

Preparing two partials of PPA1-DOX (1 mg/mol) were dissolved into the 2 mL sodium phosphate buffer (pH = 5.0) and 2 mL mouse serum (pH = 7.4), respectively, and the vials were capped and kept 37°C under nitrogen with continuously slight oscillaation. Samples (20 μL) were spiked and analyzed by HPLC under the 254 nm absorbance value after incubating 0.5 h, 1 h, 2 h, 4 h, 12 h and 24 h. For evaluation of hydrazone bond cleavage data was considered the disappearance of the major peak related to the standard curves of PPA1-DOX and DOX.

### 2.6 Immunohistochemistry

The tumor tissues were fixed in 4% paraformaldehyde, gradually dehydrated, embedded in paraffin, cut into 4um sections, and subjected for hematoxylin/eosin staining. For immuno-histochemical staining of CD4/CD8 positive cells, tissue sections processed through deparaffinage, rehydration and antigen plerosised, and endogenous peroxidase activity blockade, were incubated with mouse anti-human CD4(1:300, Proteintech, USA), mouse anti-human CD8(1:200, Proteintech, USA) at 4°C overnight, respectively. The secrions were then washed and incubated with a HRP-labeled secondary antibody at RT for 40 min. After color development through incubation with diaminobenzidine, the sections were counterstained with hematoxylin. The stained sections were observed and imaged under a light microscope.

### 2.7 Ethics Committee Approval

Animal experiments and maintenance were approved by the Laboratory Animal Ethics Committee of Shenzhen University.

### 2.8 Animal Model

Currently the institute does not provide approval or accreditation number. Six-week-old female Balb/c mice were inoculated subcutaneously with 5 × 10^6^ CT26 cells in the left armpit. The mice were randomly divided into groups with 7 mice in each group after the tumor reached about 100 mm^3^. The mice were housed in animal house of Shenzhen University. For the survival experiment, the mice were killed when the tumor reached about 1500 mm^3^. The tumor size and body weight were monitored every day after the injection of the drugs. At the end of the experiment, the tumors and major organs (heart, liver, spleen, lung, and kidney) were collected for immunohistochemistry and/or histological tests. For the PPA1-DOX distribution experiment, the tumors and major organs were subsequently analyzed with an *in vivo* imaging system (IVIS Spectrum, PerkinElmer).

### 2.9 Dosage of the Injection to Mice

DOX at 5 mg/kg, 5-Fu at 10 mg/kg, PPA1 and RAN at 15mg/kg, PPA1-DOX and RAN-DOX at 20 mg/kg were injected intraperitoneally to mice twice a week for two weeks. From the fifteen day, no further injections of the drugs were given because mice injected with 5-Fu and DOX became very sick due to toxicity. For the PPA1-DOX distribution experiment, RhB-PPA1-DOX and RhB-RAN-DOX at 20mg/kg were injected into the tail vein of the mice.

### 2.10 Statistical Analysis

All the variables were expressed as mean ± standard deviation. Student’s t-test or one-way analyses of variance (ANOVA) were performed in statistical evaluation. A p-value <0.05 was considered to be significant.

## 3 Results

### 3.1 Design of the Polypeptide-Drug Conjugate

**Figure 1:**
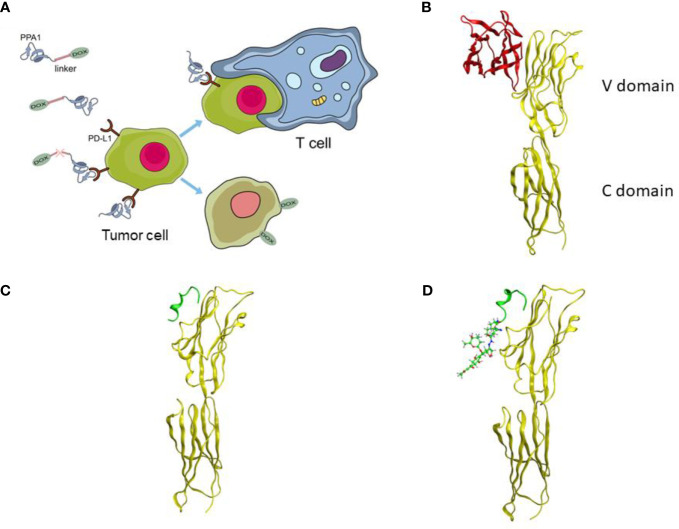
The design of the polypeptide-drug conjugate (PPA1-DOX). **(A)** The mechanism of tumor inhibition by PPA1-DOX. **(B)** The crystal structure of the PD-1/PD-L1 complex from Protein Data Bank (PDB ID: 3BIK). **(C)** The structure of PD-L1/PPA1 from molecular dynamic simulation. **(D)** The structure of PD-L1/PPA1-DOX from molecular dynamic simulation. The yellow ribbon structure stands for PD-L1; The red ribbon structure stands for PD-1; The green ribbon structure stands for PPA1; The green stick and ball molecule stands for DOX.

A tumor-specific targeted synergistic strategy was designed by coupling anti-PD-L1 polypeptide with chemotherapy for colon cancer, where a proteolysis resistant PD-L1-targeted peptide PPA1 was conjugated to DOX with a pH sensitive linker (**Figure 1A**). To verify the binding mode of PD-L1 and PD-1, we downloaded and visualized the crystal complex of PD-L1 and PD-1. PD-L1 is composed of one N-terminal V domain and one C-terminal C domain, which are joined by a short linker (22). The complex shows that PD-1 binds to the V domain of PD-L1 (**Figure 1B**). The PD-1/PD-L1 interaction could then halt or limit the development of the T cell response (23). Polypeptide PPA1 was reported to show the potential of targeting PD-L1 in colorectal cancer cell. In order to ensure the binding mode of PD-L1 and polypeptide PPA1, we performed a protein-peptide docking simulation. The results show that PPA1 possesses the PD-1 binding position, which could competitively bind to the V domain of PD-L1 (**Figure 1C**). Thus, we designed the polypeptide-drug conjugate by linking the C terminal (tail) of PPA1 and the carboxylic acid group of DOX with a linker (**Figure 1D**). Quantitatively, further binding free energy of PPA1 and PPA1-DOX showed no significant difference when binding to PD-L1.

### 3.2 Synthesis of the PD-L1-Targeted Peptide-DOX Conjugate (PPA1-DOX)

**Figure 2:**
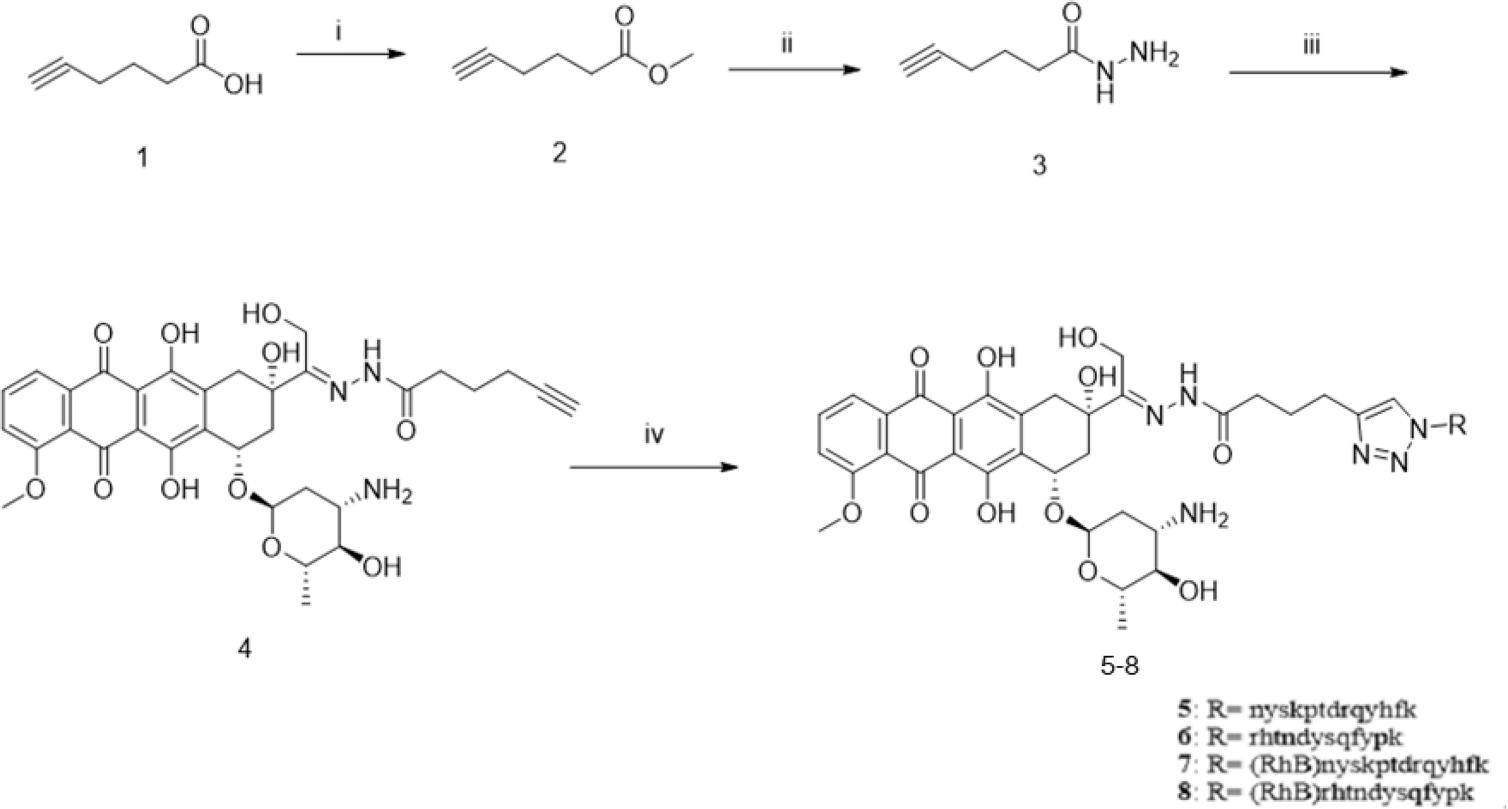
The synthesis of PPA1-DOX conjugate with an acid-labile linker. Reagents and conditions: (i) MeOH, DCC, DMAP, r.t., 4h; (ii) NH_2_-NH_2_ 80% in H_2_O, EtOH, 80°C, 6h; (iii) doxoriubicin, TFA, MeOH, 4A molecular sieves, r.t., 24h, (iv) nyskptdrqyhf-Lys(N_3_), CuSO_4_.5H_2_O, sodium ascorbate, TBTA, DMF/H_2_O, r.t., 4h, N_2_.

For the synthesis of acid-sensitive PPA1-DOX (**Figure 2**), the carboxylic acid group of 5-hexynoic acid **1** was reacted with methanol under the condensation reagent of DCC and catalysis reagent of DMAP to effectively provide the methyl 5-hexynoate **2**. Ester **2** was treated with 80% aqueous hydrazine hydrate in ethanol at 80°C for 6 hours to smoothly give acyl hydrazide **3**. The desired compound **4** with an acid-sensitive hydrazone was afforded by compound **3** coupling to commercially available doxorubicin in methanol. Compound **4** was allowed to undergo cycloaddition reaction with various peptide azides under sharpless click chemistry condition to offer the target compound **5 (**PPA1-DOX**)** in good to excellent yields. Compound **6** (RAN-DOX) was synthesized in the similar routine by using a random polypeptides. Compound **7** and **8** with rhodamine (RhB) were designed and synthesized to verify the distribution of the compounds. The intermediates were characterized by Nuclear Magnetic Resonance (NMR), including 1H-NMR, 13C-NMR, and high resolution mass spectrometry (HRMS), and the conjugates were confirmed by high performance liquid chromatography (HPLC) and HRMS. (**Supplementary Figures S1**–**S7**).

### 3.3 PPA1-DOX Conjugate Cleavages Around Tumor-Like Environment

**Figure 3:**
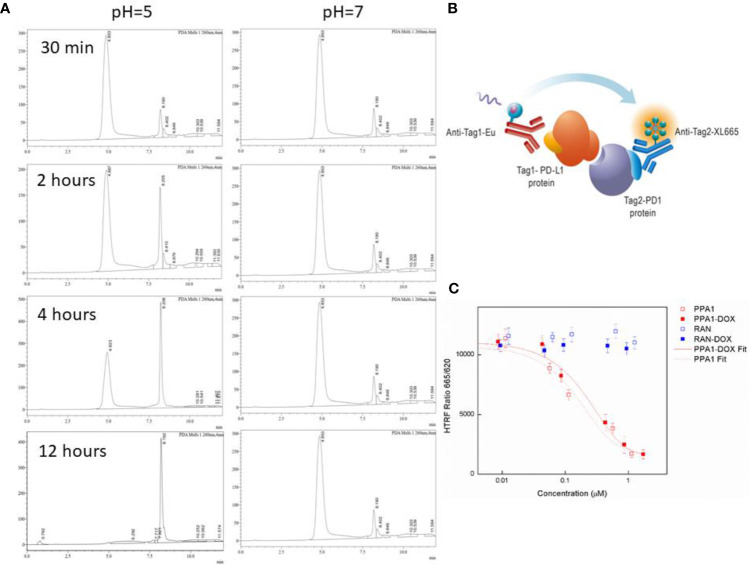
The cleavage and binding affinity of the PPA1-DOX conjugate. **(A)** Representative HPLC chromatograms of PPA1-DOX in mouse serum (pH = 7.4) upon time. Representative HPLC chromaograms of PPA1-DOX and free DOX in the weak-acid PBS buffer (pH = 5) upon time. **(B)** The mechanism of HTRF assay to test the blocking of PD-1 and PD-L1. **(C)** The result of HTRF assay of 4 kinds of drugs. Error bars indicate mean± standard deviation. n = 3.

We designed an acidic pH sensitive linker to link the polypeptide and drug. To test whether the acidic pH sensitive linker can enable the split of PPA1-DOX construct into two components around the tumor tissue, the HPLC experiment was performed (**Figures 3A, B**). When PPA1-DOX conjugate was incubated in the mouse serum (pH=7.4) at 37°C for 24 h, the HPLC result showed little change on peaks, suggesting that PPA1-DOX conjugate existed stable in nearly neutral solution. In contrast, in an acidic environment (pH=5.0), HPLC results implied that PPA1-DOX conjugate rapidly cleaved into PPA1 and DOX (**Supplementary Figure S8**). Given the neutral physiological environment around normal tissue and the acidic microenvironment around solid tumor tissues, these results thus indicate that PPA1-DOX conjugate is able to stay steady in normal tissue, while release DOX to tumor tissues due to the cleavage of acidic pH sensitive linker.

### 3.4 PPA1-DOX Conjugate Exhibits High Binding Affinity With PD-L1

The HTRF (Homogeneous Time-resolved Fluorescence) PD1/PD-L1 binding assay is designed to measure the interaction between PD1 and PD-L1 proteins. By utilizing HTRF technology, the assay enables simple and rapid characterization of compound and antibody blockers in a high throughput format (**Figure 3C**). The detailed information is listed in Material and Methods section. The HTRF data showed no significant difference in the binding affinity between free PPA1 and PPA1-DOX with PD-L1. The inhibition effect of compounds and PD-L1 was shown in **Figure 3D**, with the IC_50_ = 0.174 µM of PPA1 and IC_50_ = 0.281 µM of PPA1-DOX, respectively. The results suggested that the PPA1-DOX conjugate did not affect the interaction between PPA1 and PD-L1, which is consistent with the calculated results. Of note, both of the random polypeptide (RAN) and RAN-DOX conjugate exhibited low binding affinity with PD-L1.

### 3.5 PPA1-DOX Conjugate Shows Low Toxicity *In Vivo*

**Figure 4:**
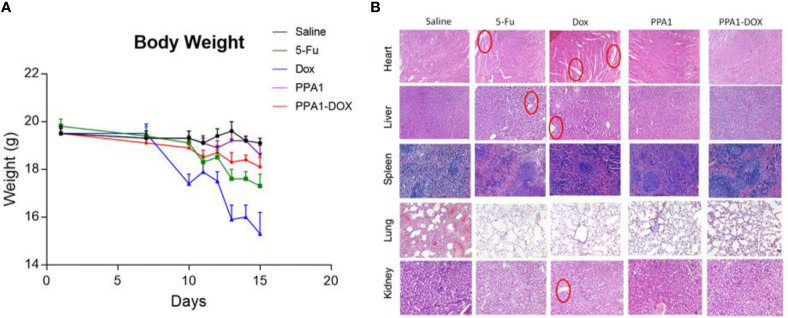
The toxicity of PPA1-DOX for treating CT26-bearing mice. **(A)** The body weight analysis of 5 groups of treatment at a dosing frequency of twice a week *via* intraperitoneal injection. **(B)** H&E staining analysis of individual tissues from 5 respective treatment groups. The cytoplasmic vacuolation and loss of myofibrillar were observed in heart damage in 5-Fu and Dox groups. For liver damage, hepatic cords loss, mild steatosis, and dilatation of blood sinus were observed in 5-Fu and Dox groups. All the damages were highlighted in red circles. Scale bars, 100 μm. The data were represented by means ± SEM. n = 7.

Since DOX can cause multi-organ toxicities in various patients, including cumulative and dose-dependent cardiotoxicity (24), we evaluated the toxicity of PPA1-DOX conjugate *in vivo*. Firstly, we measured the body weight of the tumor-bearing mice every day after 10 days of CT26 subcutaneous injection. The DOX and 5-Fu treated group showed significant weight loss after day 7, suggesting that the chemotherapeutic reagents could cause the potential systematic toxicity. In contrast, no remarkable body weight loss was observed in PPA1 and PPA1-DOX groups (**Figure 4A**). Then, the histology analysis with H&E staining was performed to evaluate the *in vivo* toxicity to major organs. The chemotherapy treated groups (DOX and 5-Fu) showed severe damages in the H&E stained sections of heart, liver and kidney, respectively (**Figure 4B**). The cytoplasmic vacuolation and loss of myofibrillar were observed in heart damage. For liver damage, hepatic cords loss, mild steatosis, and dilatation of blood sinus were observed. By contrast, PPA1-DOX and free PPA1 exhibit rather low toxicity to major organs of tumor-bearing mice.

### 3.6 PPA1-DOX Conjugate Improves Tumor-Specific Drug Delivery and Enhances Immune Response *In Vivo*

**Figure 5:**
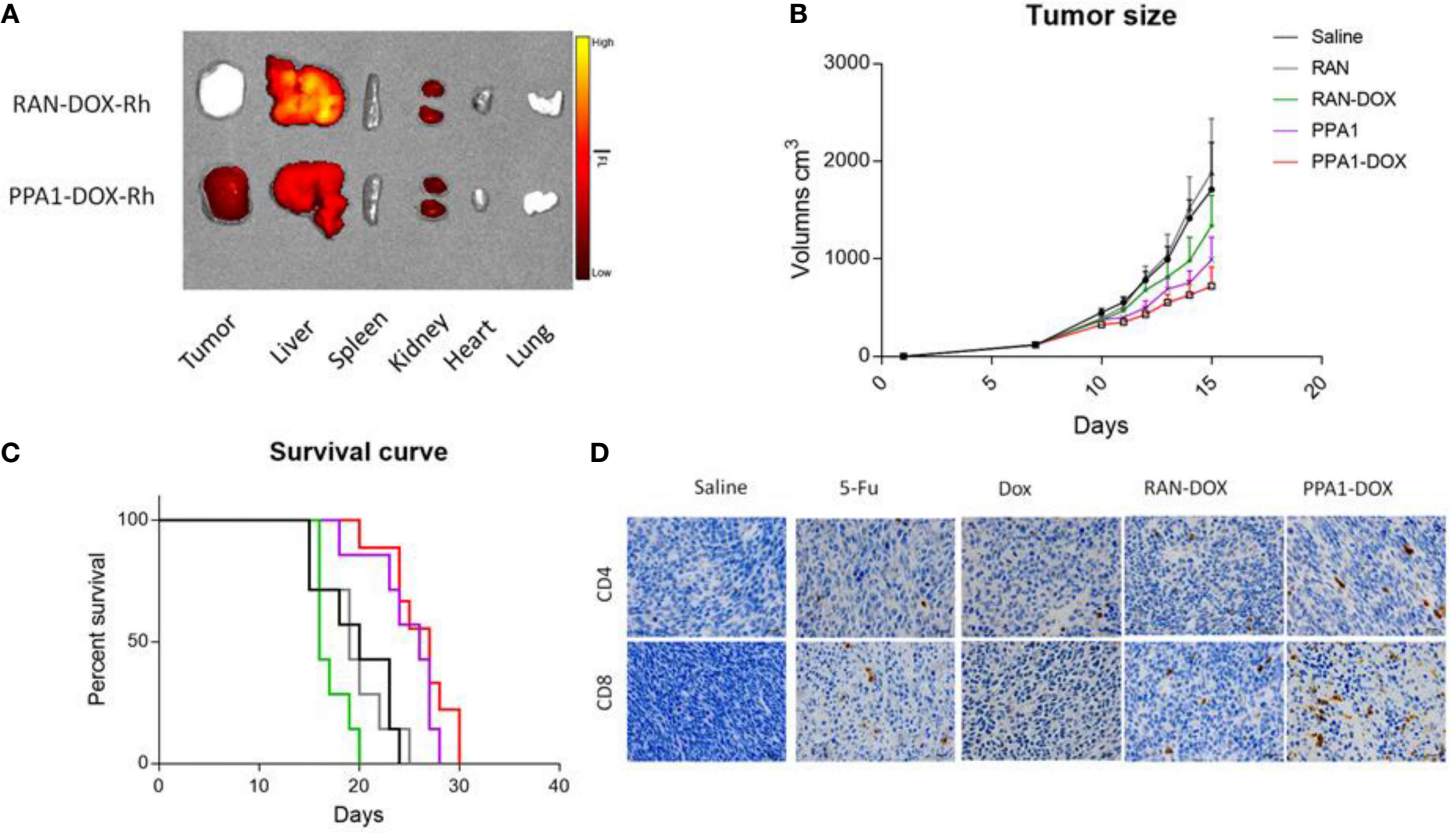
The antitumor activity of PPA1-DOX in CT26-bearing mice. **(A)** The distribution of PPA1-DOX-RhB and RAN-DOX-RhB in tumor and major tissues at 24h post-intravenous injection. **(B)** Analysis of tumor sizes from 5 respective treatment group at a dosing frequency of twice a week *via* intraperitoneal injection. **(C)** The survival curve of 5 groups of treatment at a dosing frequency of twice a week *via* intraperitoneal injection. **(D)** CD4 and CD8 immunohistochemical staining of tumor tissues from the respective treatment group. Scale bars, 50 μm. The data were represented by means ± SEM. n = 7.

Firstly, to test the specificity of tumor targeting by the PPA1-DOX conjugate, we synthesized RhB-PPA1-DOX (fluorescence of PPA1-DOX) and RhB-RAN-DOX (fluorescence of a random polypeptides-DOX) and evaluated the biodistribution of the compound in the CT26-bearing mice after 24h intravenous injection by collecting major organs for *ex vivo* fluorescence imaging. We found that the RhB signals in the tumor tissues of RhB-PPA1-DOX group were remarkably higher than those of RhB-RAN-DOX group (**Figure 5A**), while the one in liver tissues of RhB-PPA1-DOX group were significant lower than those of RhB-RAN-DOX group (**Figure 5A**), suggesting that a tumor-specific targeting was achieved by the PPA1-DOX construct. Accordingly, the improved specificity of tumor-targeted drug delivery by PPA1-DOX renders itself a significantly increased antitumor activity, as demonstrated by improved tumor growth inhibition and prolonged survival time in PPA-DOX-treated CT26 bearing mice, in comparison with RAN-DOX-treated group (**Figures 5B, C**). Interestingly, despite that PPA1-DOX and PPA1 are expected to harbour similar targeting ability in principle, the PPA1-DOX treatment led to effective suppression of tumor growth and longer survival time superior to free PPA1 treatment, most probably benefited from the cytotoxic effect of DOX once released into tumor tissues (**Figures 5B, C**).

Last but not least, to evaluate the functional reinvigoration of tumor-infiltrating lymphocytes, a sign for successful immune checkpoint blockage, we examined the functional effect of PPA1-DOX on restoring intratumoral CD4^+^ and CD8^+^ T cells by immunohistochemistry staining. We found that tumors from PPA1-DOX-treated mice showed obviously increased infiltration of both CD4^+^ and CD8^+^ T cells, as compared with RNA-DOX-, DOX-, or 5-FU-treated mice, respectively (**Figure 5D**), suggesting that PPA1-DOX can enhance immune response in colon cancer due to disruption of the inhibitory PD-1/PD-L1 immune checkpoint by the PD-L1 targeted polypeptide PPA1. The positive cell number of different groups for CD4 and CD8 were provided in **Supplementary Figure S9**.

## 4 Discussion

Severe toxic side effects and developed drug resistance are the major drawbacks of DOX in its clinical use in treating colon cancer (21, 25). Modifying the structure of DOX offers the opportunity to overcome these limitations. Recently, a novel proteolysis resistant PD-L1-targeted polypeptide, PPA1, has appeared as an outstanding tumor-targeting modification of synergistic drug conjugate for effective anti-tumor treatment (18). However, the combination regimen of coupling PD-L1 polypeptide with DOX in anti-tumor treatment has not been reported so far. In this study, we developed a novel synergistic strategy in which PPA1 was conjugated to DOX with a pH sensitive linker. Such sophisticated design could enable a tumor-specific targeted delivery of the conjugate to PD-L1 expressing tumor cells with the guidance of PPA1, as well as the controlled release of DOX to acidic tumor tissues due to the cleavage of acid-sensitive linker, thus reducing the toxicity of DOX to non-tumor tissues. In addition, given that the antitumor mechanism of DOX is its action as a topoisomerase II poison by intercalating DNA *via* its anthracycline structure (26–28) (**Supplementary Figure S10**) to inhibit DNA replication, the pH sensitive linker was designed to be connected to the azide part of DOX to reduce the steric hindrance from PPA1 (**Figure 2**). As a result, PPA1-DOX conjugate was found to exhibit a significantly lower toxicity to non-tumor cells, in particular for cardiomyocytes and hepatocytes that are major toxic targets of DOX (28, 29), and a remarkably higher antitumor activity *in vivo*, as compared with DOX and free PPA1, respectively. Of note, despite that PPA1-DOX and PPA1 are expected to harbor similar targeting ability in principle, the superiority of PPA1-DOX in tumor treatment is most likely benefited from the cytotoxic effect of DOX once released into tumor tissues, demonstrating that the combination regimen of coupling PD-L1 polypeptide with DOX represents a potential targeted treatment strategy of colon cancer.

In order to verify that the improved therapeutic efficacy and reduced toxicity of the PPA1-DOX conjugate is attributed to the tumor-targeted delivery offered by the PPA1 polypeptide, but not to the random changes in the surface modification of DOX, a random polypeptide (RAN) was conjugated to DOX as a control and the biodistribution of the fluorescence labelled compound was evaluated in the CT26-bearing mice after intravenous injection. As expected, a remarkably higher tumor-specific enrichment and lower distribution in other non-tumor major tissues was achieved by the PPA1-DOX construct, as compared with the RAN-DOX construct. Accordingly, PPA-DOX conjugate demonstrated a significantly increased antitumor activity and remarkably reduced off-target toxicity, in comparison with RAN-DOX construct, which even though showed a weak tumor inhibition effect, probably due to a few of DOX released from RAN-DOX conjugate. Thus, delivery of DOX guided by PD-L1-targeted polypeptide contributes to the improved anti-tumor effect of PPA1-DOX conjugate.

It is worth to note that the traditional drug delivery system, including DNA aptamers (11), RNA aptamers (30), peptide aptamers (12) *etc.*, could only deliver the drugs to specific target cells or proteins with little activity itself. Compared to the traditional drug delivery system, PPA1 not only acts as a targeting navigator for the drug, but also binds with PD-L1 to improve the antitumor activity of immune cells. It is well known that when PD-L1 is bound to PD-1, these ‘coinhibitory’ receptors function as breaks for the adaptive immune response to protect the host from being attacked by its own adaptive immune system, serving as immune checkpoints that effector T cells must pass to exert their functions (31). However, some cancers, including colon cancer, exploit this negative feedback loop by expressing PD-L1 to avoid being killed by T cells. Recently, antagonizing the PD-1/PD-L1 interaction has been shown to revert the exhausted phenotype of T cells and allow efficient killing of tumor cells (32, 33). In this study, binding of PD-1 by PPA1-DOX was approved *in vitro* and increased infiltration of both CD4^+^ and CD8^+^ T cells was found in tumors from PPA1-DOX treated mice. CD4^+^ and CD8^+^ T cell responses are part of the cancer immune cycle, which significantly influence the clinical treatment outcome, while the phenotype of T cell exhaustion usually occurs in both CD4+ and CD8+ T cell populations (34). Therefore, the increased CD4^+^ and CD8^+^ T cell frequencies by PPA1-DOX indicates that this conjugate is able to restore T cell function and enhance immune response in colon cancer.

The antimetabolite 5-FU remains a mainstay of standard therapy in colon cancer and is effective as a part of combination therapies that induce remissions (35). However, the chemical structure of 5-FU lacks the active synthetic site for PPA1 linker conjugation. We are working on PPA1 targeting nanoparticles, which could deliver various kinds of drugs to tumor tissues.

## 5 Conclusion

To summarize, we have designed and synthesized a novel strategy of coupling PD-L1 polypeptide with cytotoxic agent for tumor-targeted therapy in colon cancer, in which a proteolysis resistant PD-L1 targeting peptide PPA1 is conjugated with DOX by a pH sensitive linker, which could trigger the release of drugs near tumor tissues. Our data demonstrate that PPA1-DOX harbour high binding affinity with PD-L1 *in vitro* and specifically enriched within tumor when administered *in vivo*. Importantly, PPA1-DOX exhibits a significantly improved antitumor activity *in vivo*, most likely by alleviating chemotherapeutic resistance of DOX and enhancing immune response in colon cancer. Thus, targeted delivery of chemotherapeutic reagent to tumor tissues by PD-L1 polypeptide represents a potential treatment strategy of colon cancer with improved efficacy and reduced toxicity.

## Data Availability Statement

The original contributions presented in the study are included in the article/**Supplementary Material**. Further inquiries can be directed to the corresponding authors.

## Ethics Statement

The animal study was reviewed and approved by Laboratory Animal Ethics Committee of Shenzhen University.

## Author Contributions

MW, X-sS, and YY conceived the study and wrote the manuscript. ML and YZ performed experiments. YoY and XH: Data Curation. PW: Validation. ZH and JiL: Writing – Review & Editing. JuL and YiY: Supervision and Funding Acquisition. All authors contributed to the article and approved the submitted version.

## Funding

This work was supported by Shenzhen Commission of Science and Innovation programs (JCYJ20200109105613463; JCYJ20190808145211234; JCYJ20190808165003697; JCYJ20180302174121208), National Science Foundation of China (81803374; 82070978; 21807090; 81973293), Natural Science Foundation of Guangdong Province, China (Grant No. 2021A1515012436; 2020A1515010196; 2020A1515010125), SZU Medical Young Scientist Program (71201-000001), SZU Top Ranking Project (86000000210) and Medical science foundation of Guangdong province (A2017072).

## Conflict of Interest

The authors declare that the research was conducted in the absence of any commercial or financial relationships that could be construed as a potential conflict of interest.

## Publisher’s Note

All claims expressed in this article are solely those of the authors and do not necessarily represent those of their affiliated organizations, or those of the publisher, the editors and the reviewers. Any product that may be evaluated in this article, or claim that may be made by its manufacturer, is not guaranteed or endorsed by the publisher.

## Abbreviations

PD-L1, programmed death-ligand 1; ApDC, aptamer drug conjugate; DOX, doxorubicin; DMAP, 4-dimethylaminopyrid; TLC, thin-layer chromatography.
