# Supplementary material for: A Novel Strategy Conjugating PD-L1 Polypeptide With Doxorubicin Alleviates Chemotherapeutic Resistance and Enhances Immune Response in Colon Cancer
Source: Front Oncol. 2021 Nov 10;11:737323. doi: 10.3389/fonc.2021.737323 (PMC8631515; doi:10.3389/fonc.2021.737323)
Supplement: Supplementary file 1 [file DataSheet_1.docx]

**Supporting Information**

**A Novel Strategy Conjugating PD-L1 Polypeptide with Doxorubicin** **Alleviates Chemotherapeutic Resistance and Enhances Immune Response in Colon Cancer**

**Supporting figures**


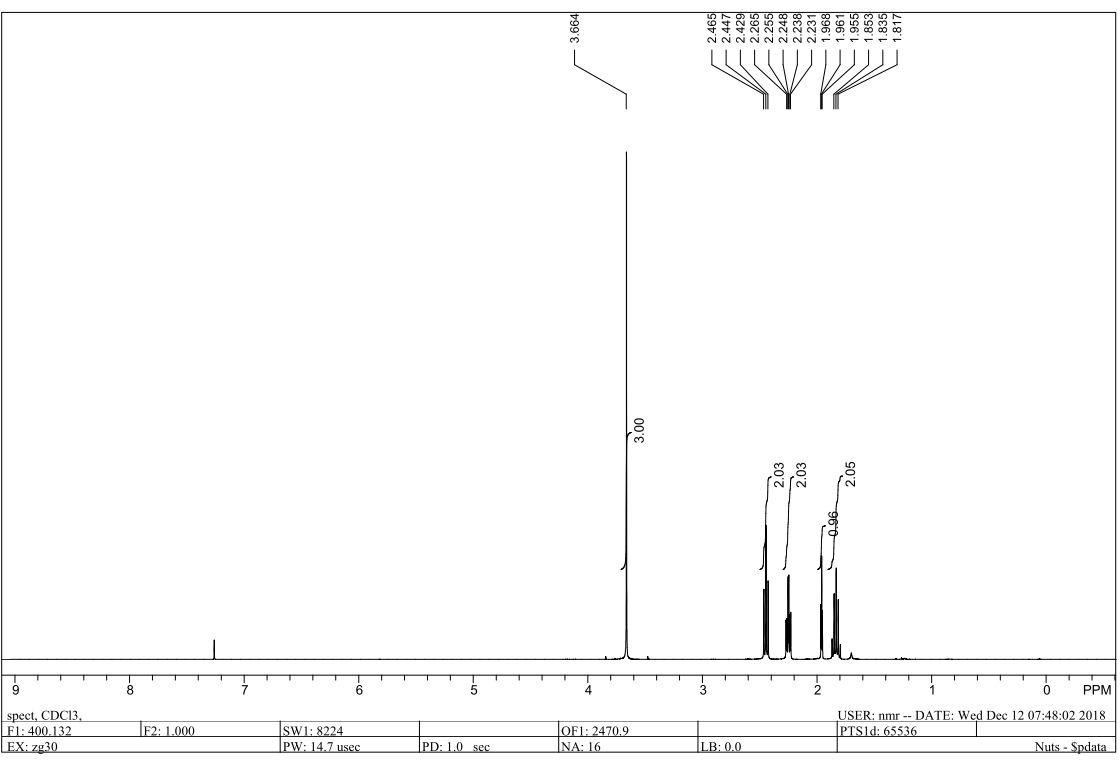


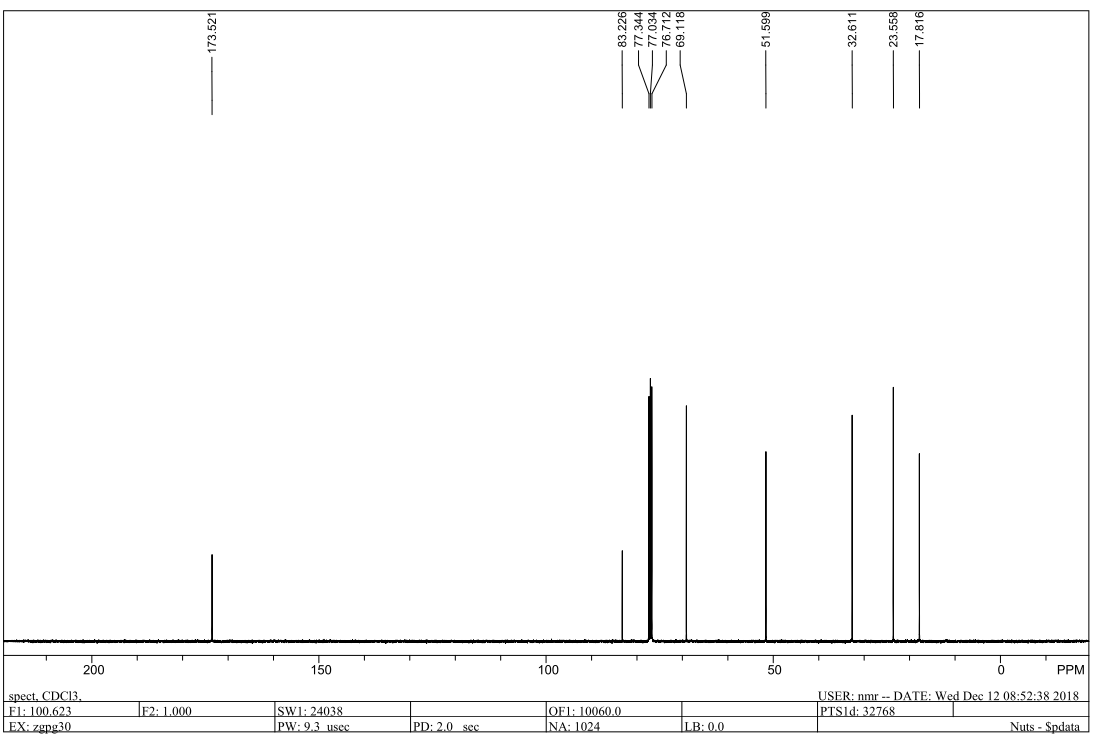


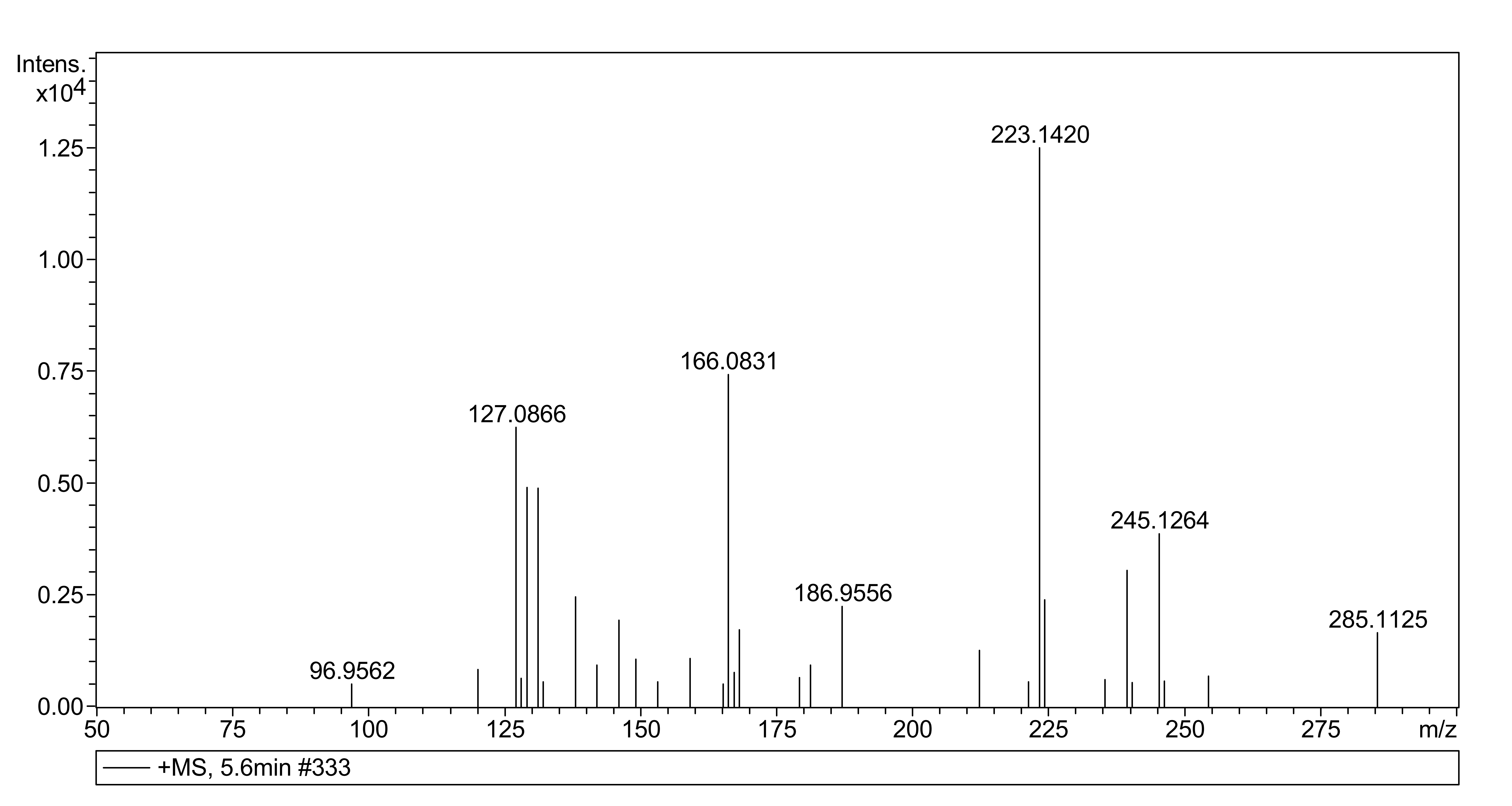


**Supplementary Figure 1: ^1^H-NMR, ^13^C-NMR and MS spectra of compound 2**

^1^H NMR (400 MHz, Chloroform-*d*) δ 3.66 (s, 3H), 2.45 (t, *J* = 7.4 Hz, 2H), 2.25 (td, *J* = 6.9, 2.6 Hz, 2H), 1.96 (t, *J* = 2.6 Hz, 1H), 1.90 – 1.76 (m, 2H). ^13^C NMR (101 MHz, CDCl_3_) δ 173.53, 83.25, 69.15, 51.63, 32.65, 23.59, 17.85. HRMS calcd. for [C_7_H_10_O_2_+H]^+^127.0754, found 127.0866.


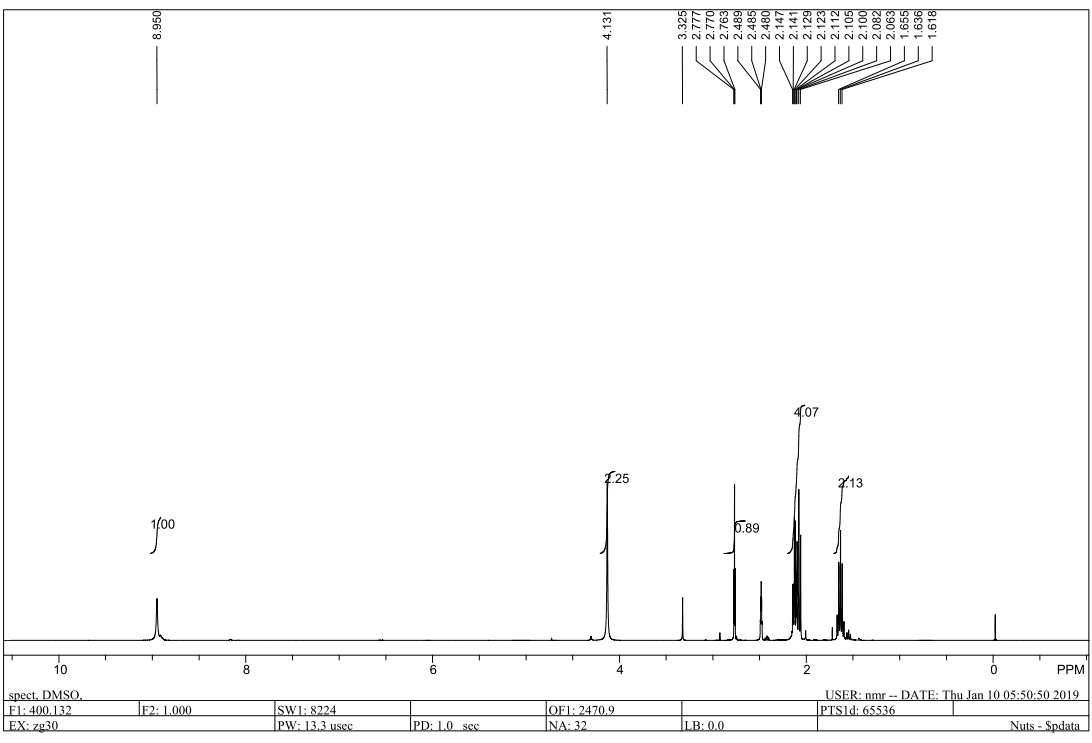

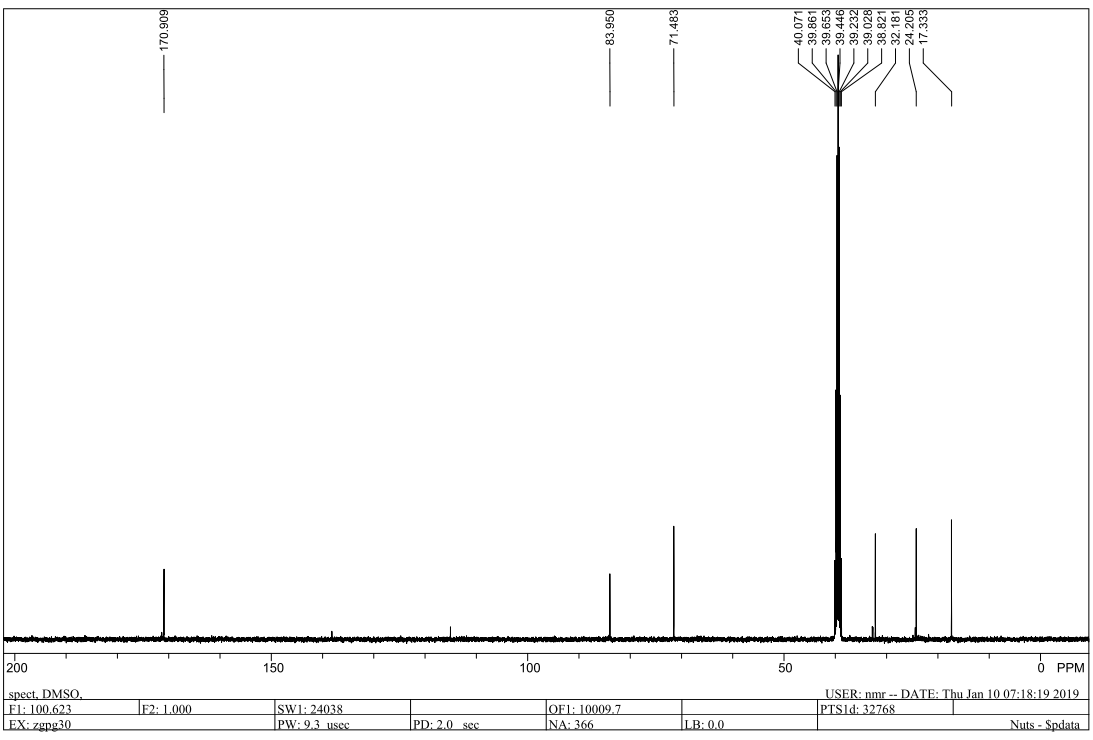


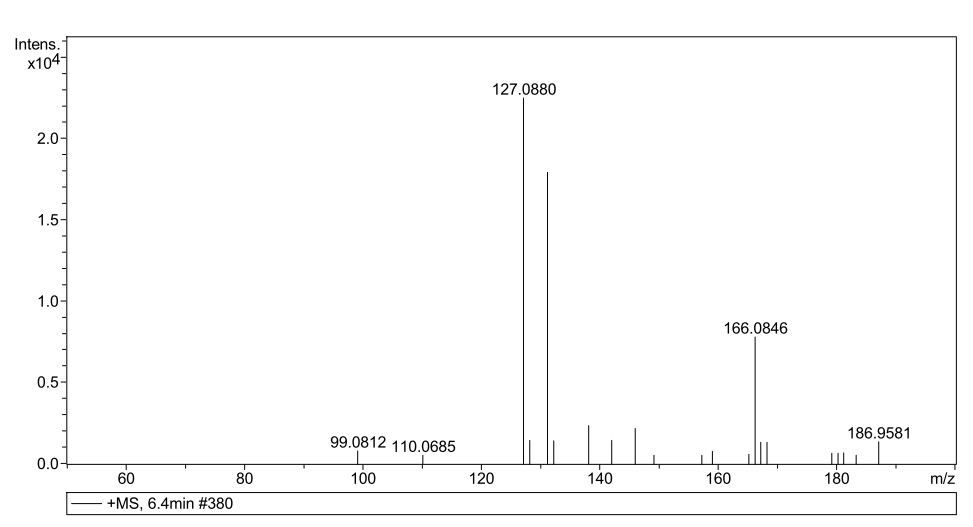


**Supplementary Figure 2: ^1^H-NMR, ^13^C-NMR and MS spectra of compound 3**

^1^H NMR (400 MHz, DMSO-*d*_6_) δ 8.95 (s, 1H), 2.77 (t, *J* = 2.6 Hz, 1H), 2.17 – 2.04 (m, 4H), 1.70 – 1.58 (m, 2H). ^13^C NMR (101 MHz, DMSO) δ 171.36, 170.93, 138.18, 115.07, 83.98, 71.51, 32.81, 32.71, 32.22, 24.44, 24.24, 17.37. HRMS calcd. for [C_6_H_10_N_2_O+H]^+^127.0866, found 127.0880.


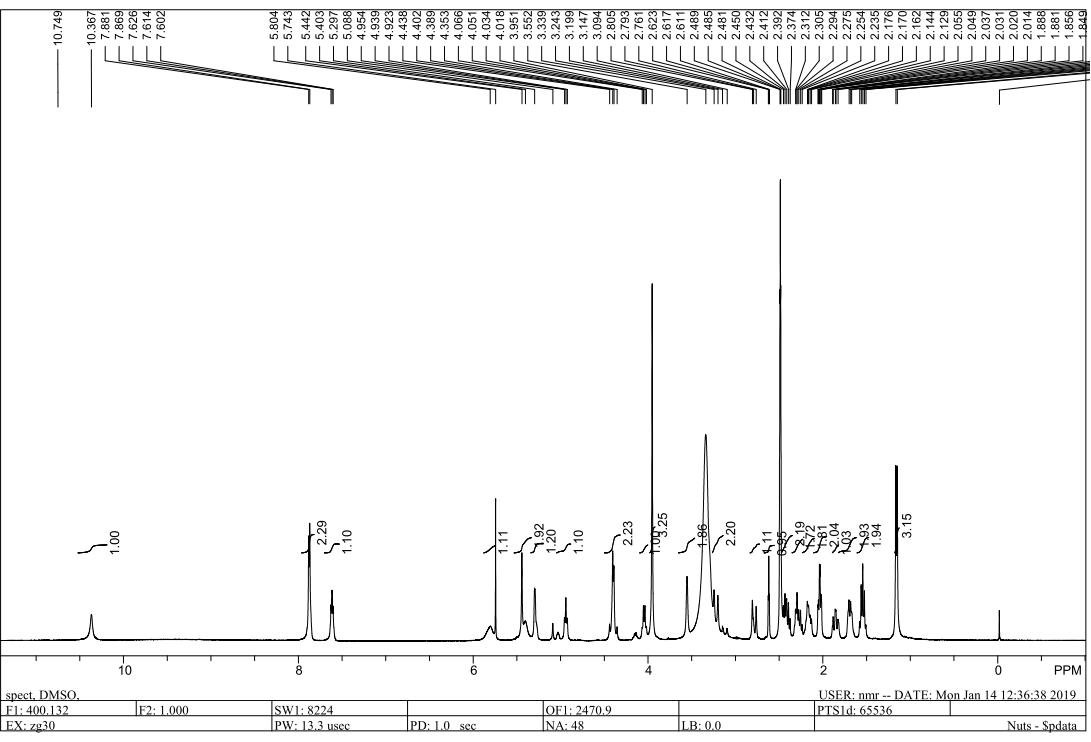


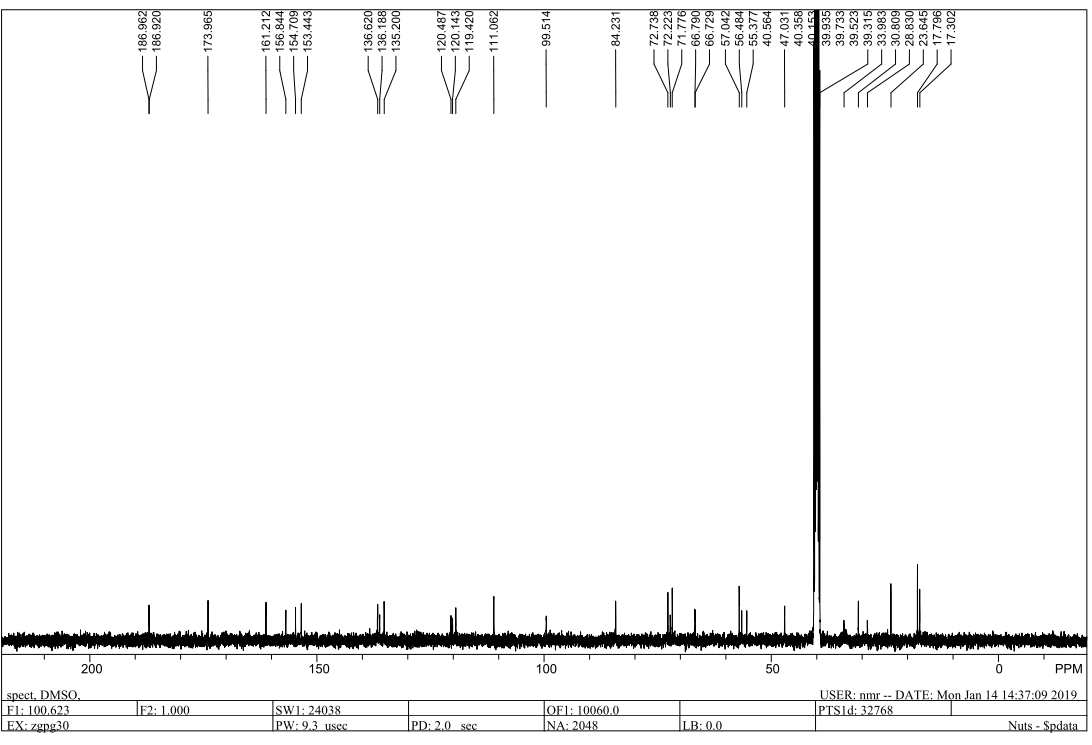


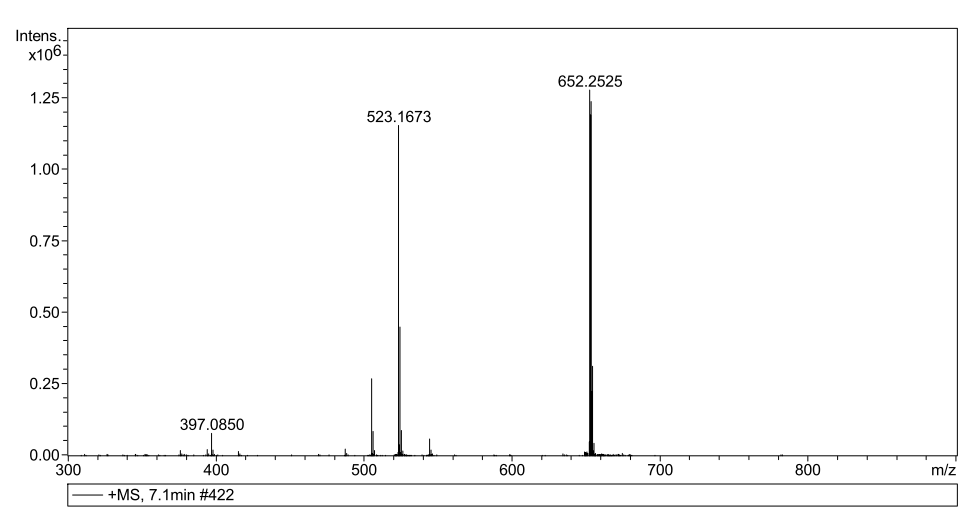


**Supplementary Figure 3: ^1^H-NMR, ^13^C-NMR and MS spectra of compound 4**

^1^H NMR (400 MHz, DMSO-*d*_6_) δ 10.37 (s, 1H), 7.87 (t, *J* = 4.9 Hz, 2H), 7.61 (p, *J* = 4.0 Hz, 1H), 5.81 (s, 1H), 5.44 (s, 2H), 5.40 (s, 1H), 4.94 (t, *J* = 6.6 Hz, 1H), 4.46 – 4.33 (m, 2H), 4.04 (q, *J* = 6.6 Hz, 1H), 3.95 (s, 3H), 3.55 (s, 2H), 3.22 (d, *J* = 17.4 Hz, 2H), 2.83 – 2.74 (m, 1H), 2.62 (t, *J* = 2.7 Hz, 1H), 2.41 (dt, *J* = 15.6, 7.3 Hz, 3H), 2.35 – 2.11 (m, 4H), 2.03 (td, *J* = 7.2, 2.7 Hz, 2H), 1.85 (td, *J* = 12.8, 3.7 Hz, 1H), 1.69 (q, *J* = 6.1, 5.5 Hz, 2H), 1.54 (p, *J* = 7.4 Hz, 2H), 1.16 (d, *J* = 6.5 Hz, 3H). ^13^C NMR (101 MHz, DMSO) δ 186.97, 186.93, 173.98, 161.22, 156.86, 154.72, 153.46, 136.64, 136.21, 135.22, 120.51, 120.16, 119.44, 111.08, 99.53, 84.26, 72.77, 72.25, 71.81, 66.82, 57.07, 56.52, 55.41, 47.07, 34.02, 30.85, 28.86, 23.68, 17.83, 17.34. HRMS calcd. for [C_33_H_37_N_3_O_11_+H]^+^652.2501, found 652.2525.


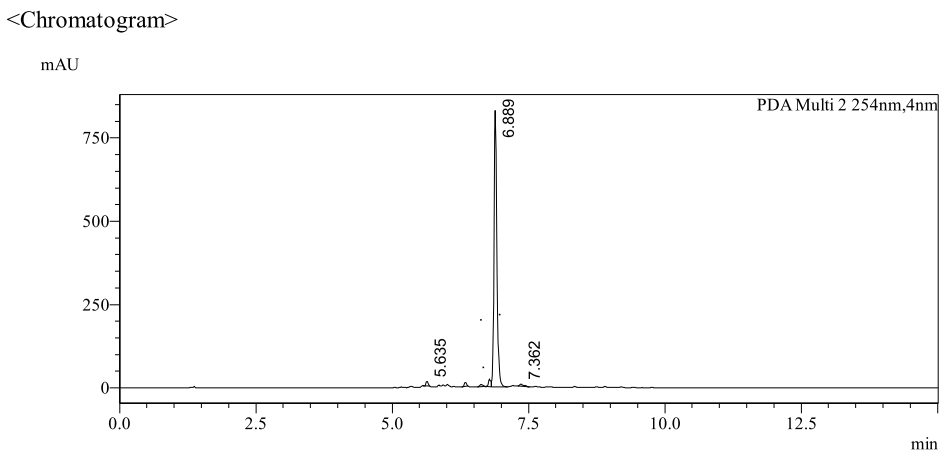


a


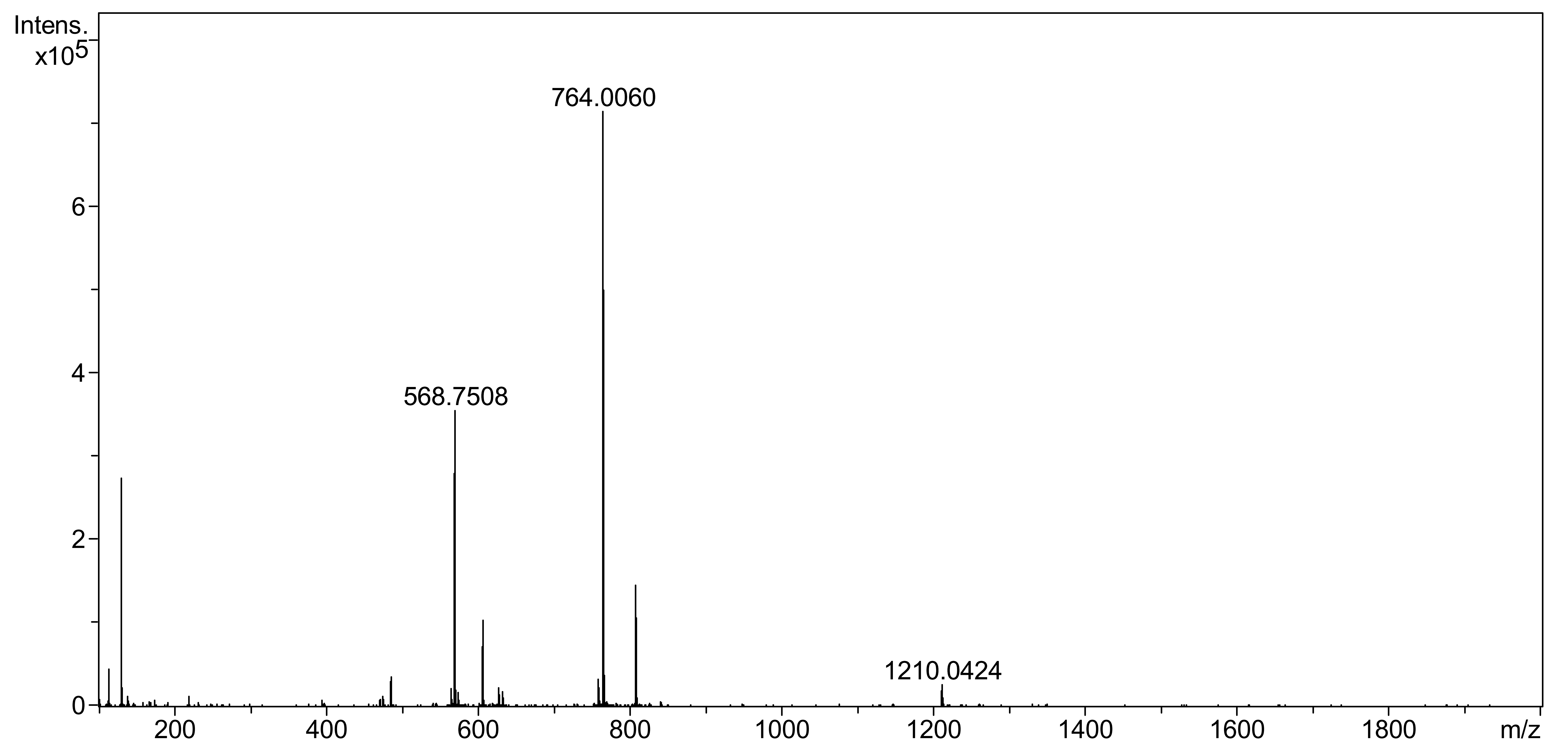


b

**Supplementary Figure 4: HPLC and MS spectra of conjugate 5 (PPA1-DOX)**

(a) The HPLC chromatogram of the PPA1-DOX conjugate. (b) HRMS (ESI): calculated for [C_111_H_148_N_28_O_34_+H]^+^ 2418.0786, found [M+2H]^2+^:1210.0424.


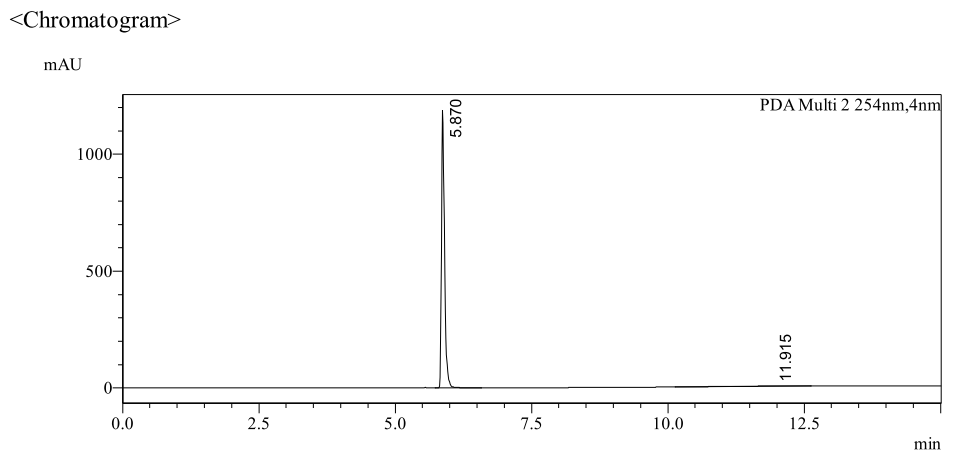


a


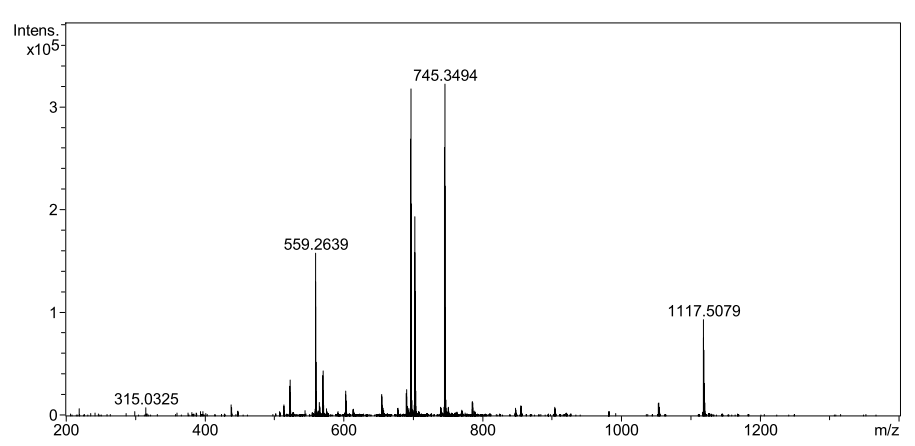


b

**Supplementary Figure 5 : HPLC and HRMS spectra of conjugate 6 (RAN-DOX)**

(a) The HPLC chromatogram of the PPA1-DOX conjugate. (b) HRMS (ESI): calculated for [C_103_H_133_N_25_O_32_+H]^+^ 2232.9621, found [M+2H]^2+^:1117.5079, found [M+3H]^3+^: 745.3494, found [M+4H]^4+^: 559.2639.


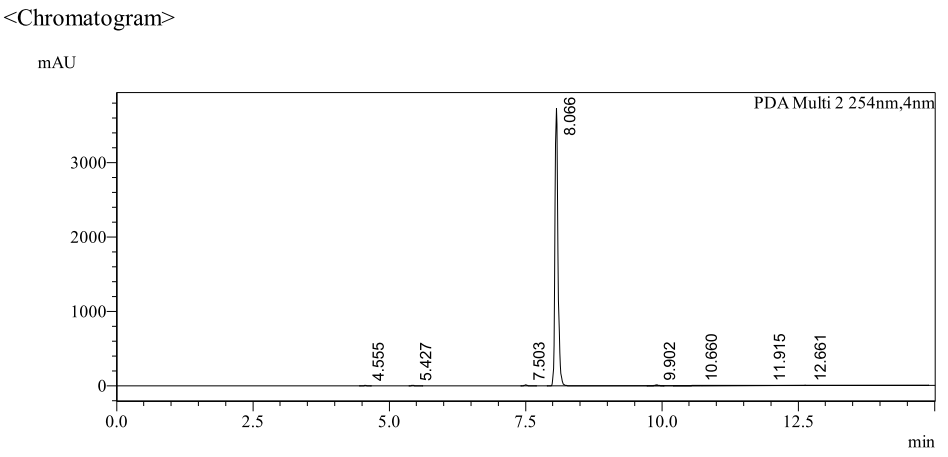


a


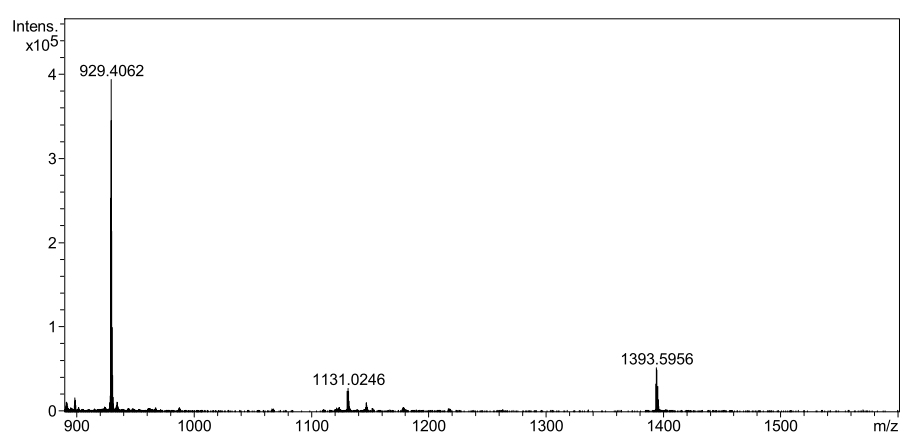


b

**Supplementary Figure 6 : HPLC and MS spectra of conjugate 7 (RhB-PPA1-DOX)**

(a) The HPLC chromatogram of the PPA1-DOX conjugate. (b) HRMS (ESI): calculated for [C_137_H_174_N_29_O_35_+H]^+^ 2785.2722, found [M+2H]^2+^:1393.5956, found [M+3H]^3+^: 929.4062.


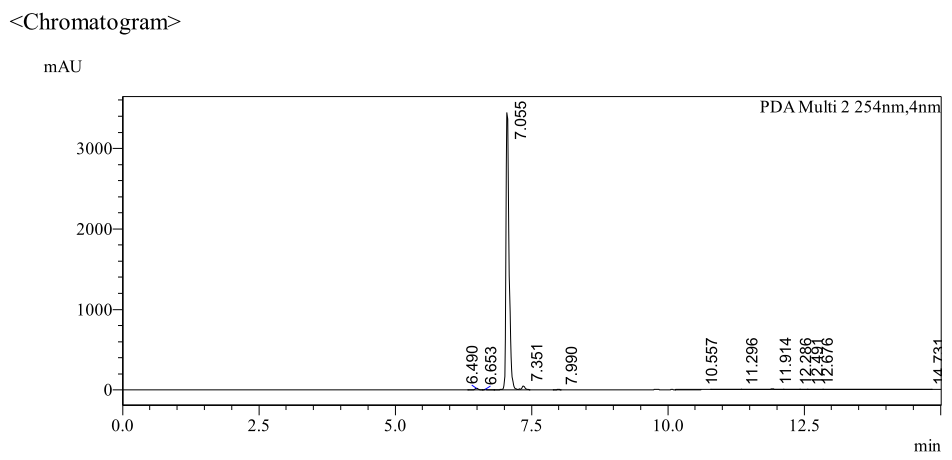


a


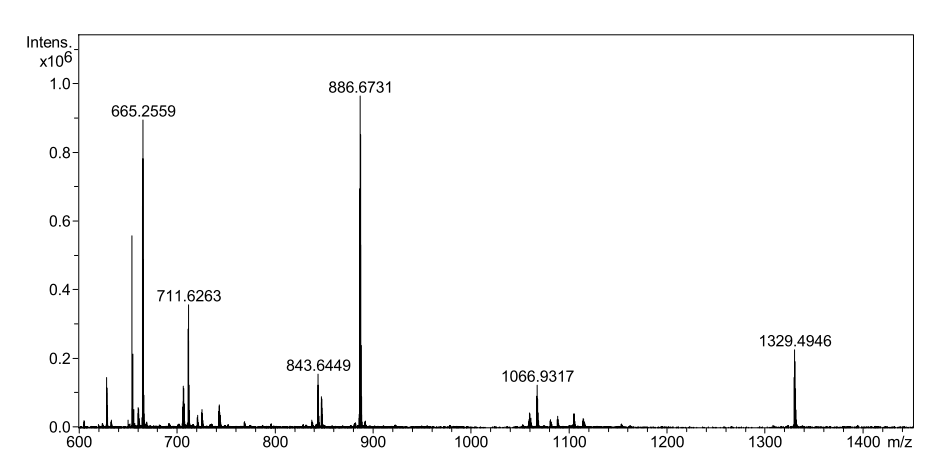


b

**Supplementary Figure 7: HPLC and MS spectra of conjugate 8 (RhB-RAN-DOX)**

1. The HPLC chromatogram of the PPA1-DOX conjugate. (b) HRMS (ESI): calculated for [C_131_H_162_N_27_O_34_+H]^+^ 2658.1845, found [M+2H]^2+^:1329.4946, found [M+3H]^3+^: 886.6731, found [M+4H]^4+^: 665.2559.


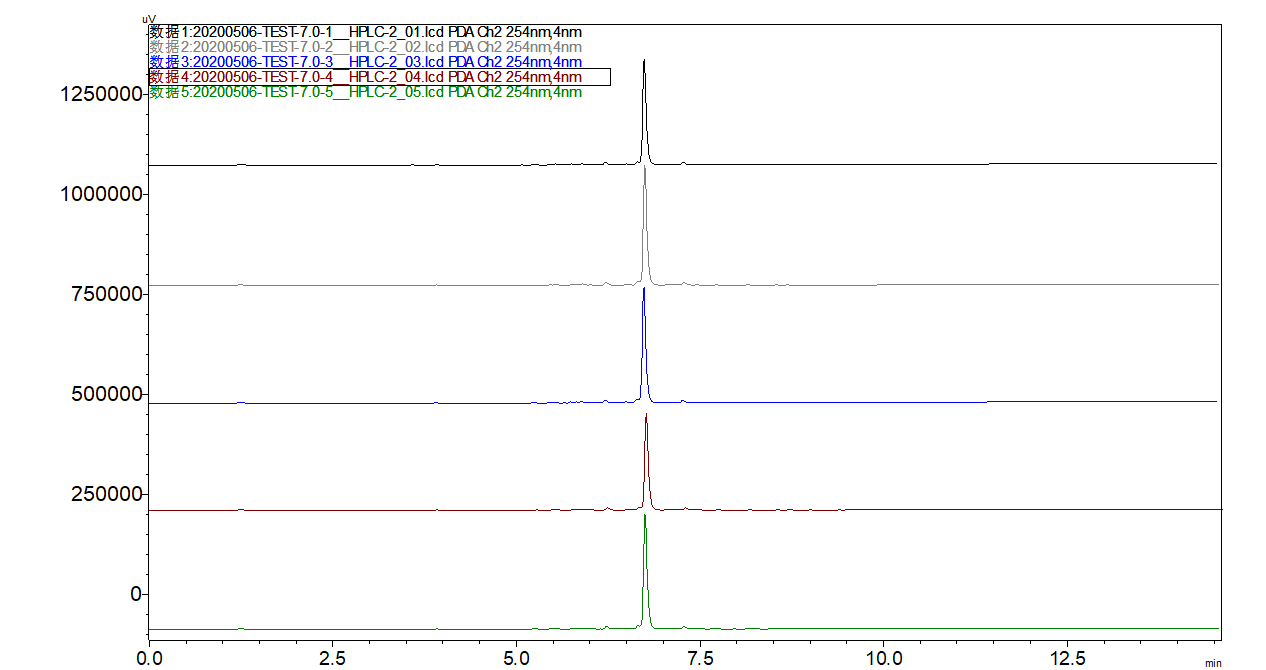


PPA1-DOX

a


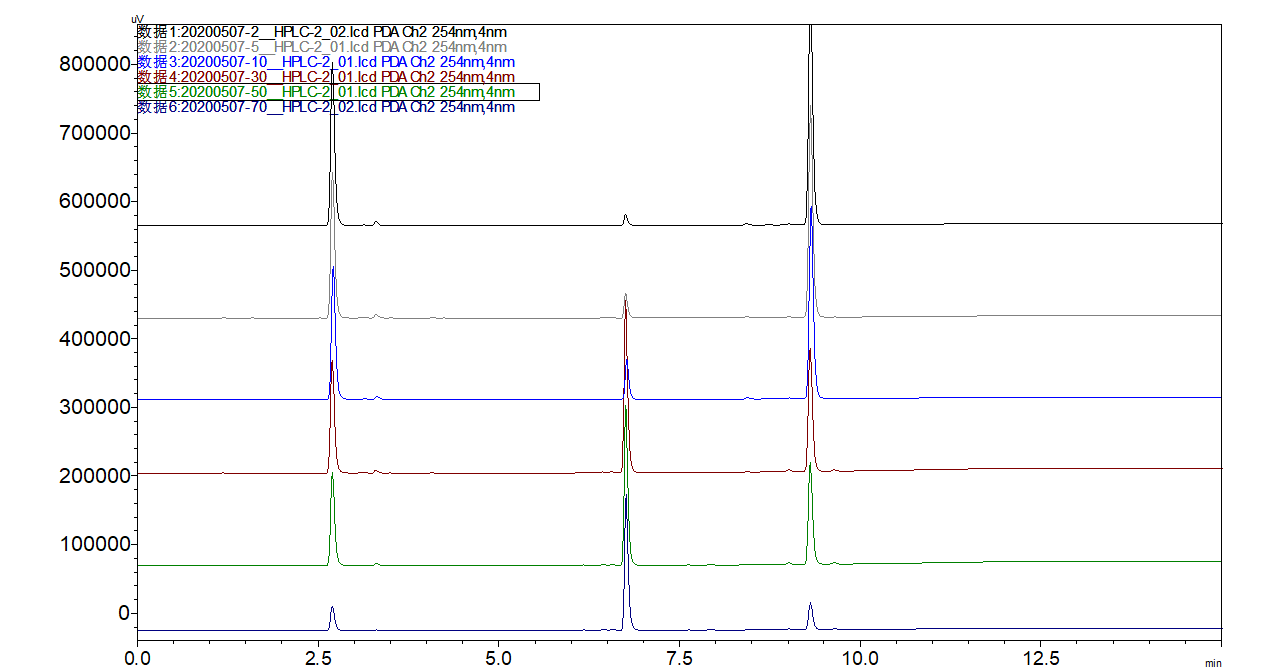


PPA1

DOX

PPA1-DOX

b

**Supplementary Figure 8 ：The stabilization of PPA1-DOX in the mouse serum and the release of free DOX in the weak acid environment detected by HPLC. (a)** The standardized concentration of PPA1-DOX in mouse serum (pH=7.4) upon time. **(b)** The standardized concentration of PPA1-DOX in the weak-acid PBS buffer (pH=5) upon time.


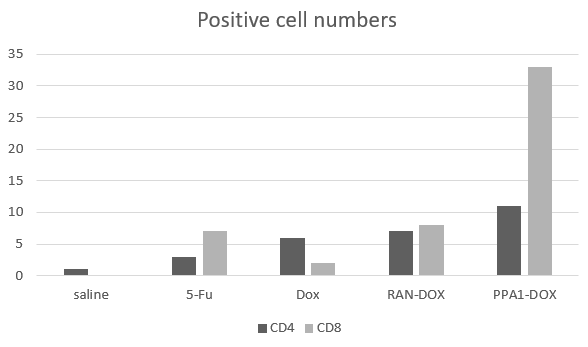


**Supplementary Figure 9：The positive cell number of different groups for CD4 and CD8. The cell number was counted by imageJ software.**


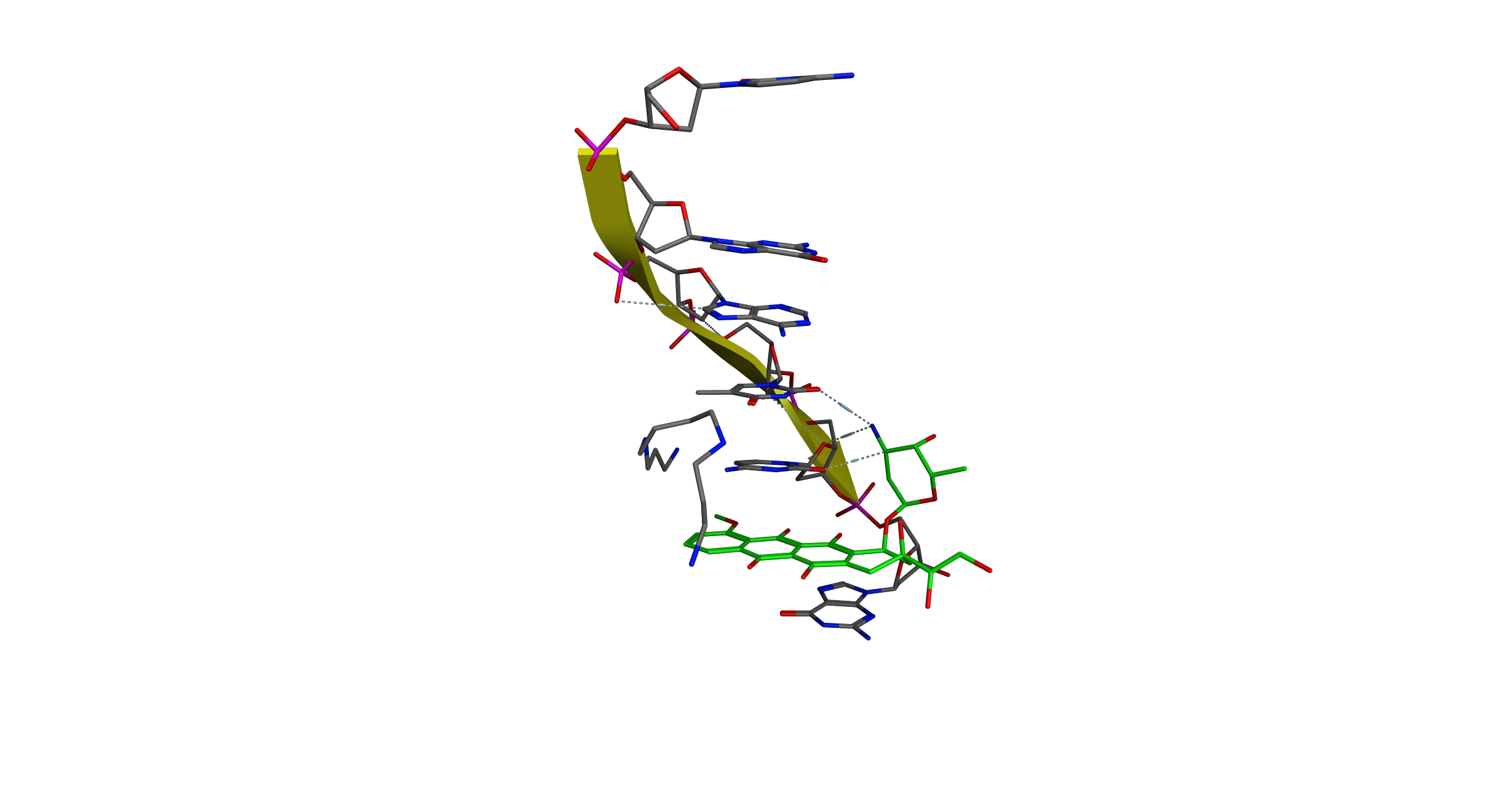


**Supplementary Figure 10：The interaction of DOX and DNA calculated by Autodock and pyMOL. PDB ID: 1D12. The green compound is DOX and the other components are DNA scaffold.**
